# Statin-Induced Geranylgeranyl Pyrophosphate Depletion Promotes Ferroptosis-Related Senescence in Adipose Tissue

**DOI:** 10.3390/nu14204365

**Published:** 2022-10-18

**Authors:** Xin Shu, Jiaqi Wu, Tao Zhang, Xiaoyu Ma, Zuoqin Du, Jin Xu, Jingcan You, Liqun Wang, Ni Chen, Mao Luo, Jianbo Wu

**Affiliations:** 1 Drug Discovery Research Center, Southwest Medical University, Luzhou 646000, China; 2 Laboratory for Cardiovascular Pharmacology, Department of Pharmacology, School of Pharmacy, Southwest Medical University, Luzhou 646000, China; 3 Metabolic Vascular Disease Key Laboratory of Sichuan Province, Southwest Medical University, Luzhou 646000, China; 4 Luzhou Municipal Key Laboratory of Thrombosis and Vascular Biology, Southwest Medical University, Luzhou 646000, China

**Keywords:** obesity, statin, senescence, ferroptosis, adipose tissue

## Abstract

Statin treatment is accepted to prevent adverse cardiovascular events. However, atorvastatin, an HMG-CoA reductase inhibitor, has been reported to exhibit distinct effects on senescent phenotypes. Whether atorvastatin can induce adipose tissue senescence and the mechanisms involved are unknown. The effects of atorvastatin-induced senescence were examined in mouse adipose tissue explants. Here, we showed that statin initiated higher levels of mRNA related to cellular senescence markers and senescence-associated secretory phenotype (SASP), as well as increased accumulation of the senescence-associated β-galactosidase (SA-β-gal) stain in adipose tissues. Furthermore, we found that the levels of reactive oxygen species (ROS), malondialdehyde (MDA), and Fe^2+^ were elevated in adipose tissues treated with atorvastatin, accompanied by a decrease in the expression of glutathione (GSH), and glutathione peroxidase 4 (GPX4), indicating an iron-dependent ferroptosis. Atorvastatin-induced was prevented by a selective ferroptosis inhibitor (Fer-1). Moreover, supplementation with geranylgeranyl pyrophosphate (GGPP), a metabolic intermediate, reversed atorvastatin-induced senescence, SASP, and lipid peroxidation in adipose tissue explants. Atorvastatin depleted GGPP production, but not Fer-1. Atorvastatin was able to induce ferroptosis in adipose tissue, which was due to increased ROS and an increase in cellular senescence. Moreover, this effect could be reversed by the supplement of GGPP. Taken together, our results suggest that the induction of ferroptosis contributed to statin-induced cell senescence in adipose tissue.

## 1. Introduction

Statins inhibit 3-hydroxy-3-methylglutaryl-CoA reductase (HMGCR), reducing cholesterol biosynthesis and related cardiovascular events [1]. Growing evidence indicates that both senescence and their secretory pro-inflammatory factors, the senescence-associated secretory phenotype (SASP), is connected to metabolic dysfunction. During obesity, the accumulation of senescent cells in adipose tissues has causally resulted in insulin resistance. A previous report indicated that obesity causes adipose tissue dysfunction, associated with adipocyte senescence, leading to impaired adipogenesis and the secretion of inflammatory cytokine. The cellular senescence of adipose tissues has been linked to mitochondrial dysfunction and abnormal change in reactive oxygen species (ROS) [2,3,4].

Statins exhibit distinct effects on senescent phenotypes in the different drug and cell types. Simvastatin can reduce SASP-related pro-inflammatory cytokines such as interleukin-6 (IL-6), interleukin-8 (IL-8), and monocyte chemoattractant protein-1 (MCP-1) [5,6]. The treatment of pravastatin could prevent protease inhibitors (PI)-induced cell cycle arrest and SA-β-gal activity from mesenchymal stem cells and chondrocytes [7,8]. Similarly, atorvastatin, or mevastatin dose-dependently inhibited the onset of endothelial progenitor cells senescence, which is involved in the transcriptional regulation of multiple cell cycle regulatory proteins [9]. However, atorvastatin also appears to induce SA-β-gal expression and retard growth by myofibroblasts [6], low dose lovastatin induced senescence, and G1 cell cycle arrest in human prostate cancer cells [10]. Moreover, atorvastatin attenuates adipocyte maturation by inhibiting isoprenoid biosynthesis, and impairs glucose tolerance [11,12], but it is unknown whether statins affect the senescence in adipose tissue.

Although statins regulate senescence by a robust cell cycle arrest and the induction of a pro-inflammatory SASP, the underling mechanism remains unknown. No studies have implicated statins in mediating ferroptosis signaling pathways that initiate senescence in adipose tissues. Statins regulate the mevalonate (MVA) pathway, including the isoprenoids geranylgeranyl pyrophosphate (GGPP) and farnesyl pyrophosphate (FPP). It was reported that supplementation of GGPP significantly prevented atorvastatin-induced inhibition of endothelial progenitor cells senescence [9]. Ferroptosis is a new form of iron-dependent lipid peroxidation during the cell death process, including depletion of glutathione (GSH) and subsequently glutathione peroxidase 4 (GPX4) inactivation [13,14], followed by an accumulation of reactive oxygen species (ROS) and consequent cell death [15].

A number of studies have provided direct evidence for the association between ferroptosis and senescence [16,17,18]. In contrast, a recent study showed that senescent cells are highly resistant to ferroptosis due to intracellular iron accumulation [19]. In adipose tissue, iron abnormality has been related to insulin resistance and obesity-related disorders [20,21]. However, the relationship among statin, ferroptosis, and senescence, has not been reported in adipose tissues. We investigated the effect of atorvastatin on the senescence markers and found a high dose of atorvastatin could induce adipose tissue senescence (P16, P21, and P53) and SASP, including PAI-1, MMP3, CD68, IL-6, MCP-1, and IGF-1. Atorvastatin significantly increased ROS, Fe^2+^, and MDA levels, while the GSH content was decreased in adipose tissues. In further analysis, we demonstrated that GPX4 was decreased by the treatment of atorvastatin. The supplementation of GGPP restores atorvastatin-induced senescence, SASP, and lipid peroxidation. GGPP and ferroptosis inhibitor can alleviate atorvastatin-induced lipid peroxidation in adipose tissues. Our results indicate that statin-induced senescence is associated with ferroptosis in adipose tissues.

## 2. Results

### 2.1. Statins Induce Senescence in Adipose Tissue 

**Figure 1:**
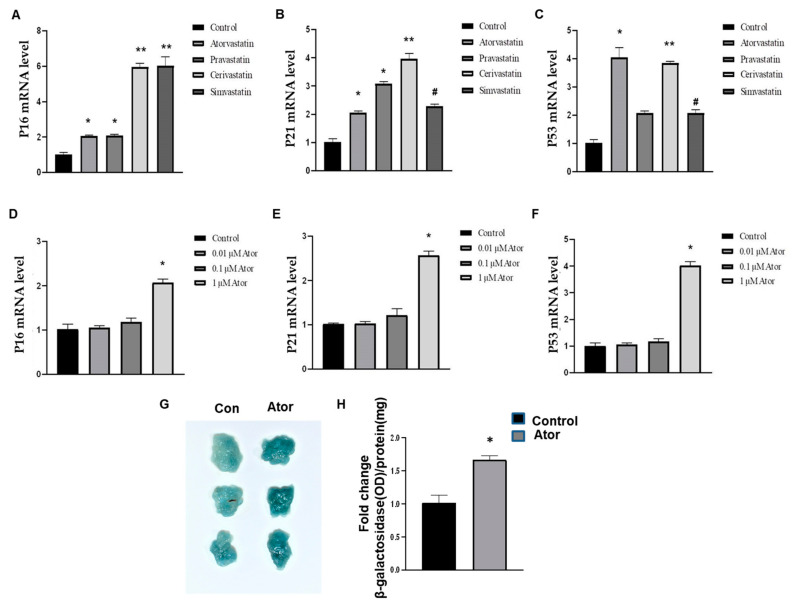
Statins induce the onset of senescence in adipose tissue. (**A**–**C**). Isolated epidydimal adipose tissues (EAT) were treated with statins (1 μM) for 18 h, as indicated. qPCR analysis of total RNA isolated from EAT for P16 (**A**), P21 (**B**), and P53 (**C**) mRNAs, respectively. Data were normalized to the amount of 18S mRNA and expressed relative to the corresponding control. *n* = 6 per group. * *p* < 0.05 vs. control; ** *p* < 0.05 vs. control; ^#^ *p* < 0.05 vs. control. Data are mean ± SEM. (**D**–**F**). EATs were treated with atorvastatin (0, 0.01, 0.1, 1 μM) for 18 h, as indicated. qPCR analysis of total RNA isolated from EAT for P16 (**D**), P21 (**E**), and P53 (**F**) mRNAs, respectively. Data were normalized to the amount of 18S mRNA and expressed relative to the corresponding control. *n* = 6 per group. * *p* < 0.05 vs. control, 0.01, 0.1 μM. Data are mean ± SEM. Ator, Atorvastatin. (**G**,**H**). Senescence was evaluated in terms of SA-β-galactosidase activity and expressed as the ratio of tissue protein (mg). *n* = 6 per group. * *p* < 0.05 vs. control. Data shown as mean ± SEM.

We first studied the concentration (1 μM) of statin needed to induce senescence in adipose tissue explants. The mRNA levels of cell cycle genes (P16, P21, and P53) were evaluated by qPCR in adipose tissue explants after 18 h of statin treatment. As treated by statins, the induction of mRNA levels was significantly higher in adipose tissue compared with control group. Cerivastatin and fluvastatin had the most potent induction of mRNA in the levels of P16, P21, and P53, respectively (Figure 1A–C). Atorvastatin is a well-studied statin with high potency to lower LDL cholesterol compared with other statins, such as simvastatin, pravastatin, lovastatin, and fluvastatin [22,23,24]. Therefore, all the subsequent experiments we performed by using atorvastatin.

In an attempt to clarify the effect of atorvastatin on senescence, adipose tissue explants were exposed to different concentrations of atorvastatin for 18 h (0.01 μM–1 μM), which induces the expression of P16, P21, and P53 at 1 μM, when compared to the other concentrations and control (Figure 1D–F). In addition, the treatment of atorvastatin at 1 μM decreased the SA-β-gal activity in adipose tissue explants compared with control (Figure 1G,H). Therefore, it is postulated that a high dose of statins could induce adipose tissue senescence. Thus, the effects of statins appear to be associated with both concentration and cell type for senescence. Our results suggest that atorvastatin induces senescence in adipose tissues in dose-dependent manner.

### 2.2. Supplementation of GGPP Restores Atorvastatin-Induced Senescence and SASP

**Figure 2:**
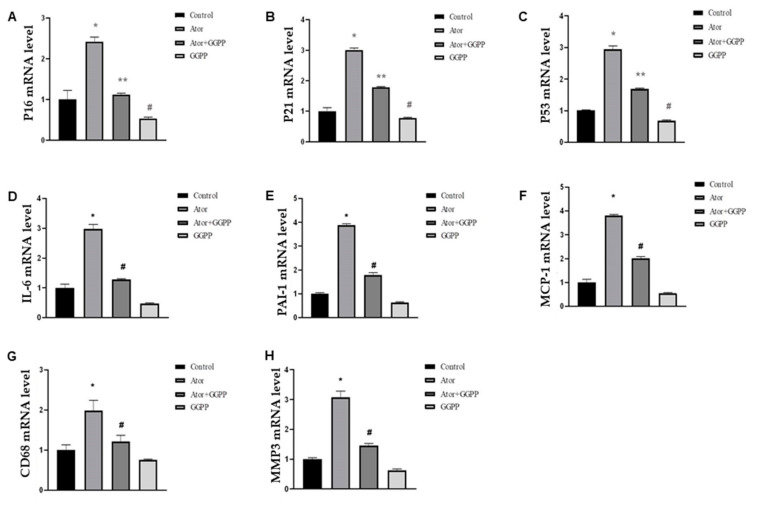
Supplementation of GGPP restores atorvastatin-induced senescence. (**A**–**C**). EAT explants were treated with atorvastatin (1 μM) plus supplementation with and without GGPP (50 μM) as indicated. qPCR analysis of total RNA isolated from EAT for P16 (**A**), P21 (**B**), and P53 (**C**) mRNAs, respectively. Data were normalized to the amount of 18S mRNA and expressed relative to the corresponding control. *n* = 6 per group. * *p* < 0.05 vs. control. ** *p* < 0.05 vs. Ator; ^#^ *p* < 0.05 vs. Control; (**D**–**H**). The mRNA levels of MCP-1, MMP3, IL-6, PAI-1, CD68, and MMP3 of EAT were quantified by qPCR. * *p* < 0.05 vs. Control; ^#^ *p* < 0.05 vs. Ator. Data are mean ± SEM. Ator, atorvastatin.

Statins catalyze the synthesis of mevalonate and reduce synthesis of geranylgeranyl pyrophosphate (GGPP). To assess whether atorvastatin-induced senescence is due to GGPP depletion, we added 50 μM GGPP in adipose tissue explants and conducted qPCR in the absence or presence of atorvastatin. GGPP treatment led to a complete reversal of P16 mRNA and most P21, P53 mRNA levels (Figure 2A–C). Interestingly, GGPP alone showed significant inhibition in the levels of P16, P21, and P53 mRNA, which is likely related to obesity-induced senescence in HFD mice [25]. Similarly, we found that atorvastatin increased the mRNA levels of SASP-specific markers, including PAI-1, MMP3, CD68, IL-6, MCP-1, and IGF-1 in adipose tissue explants (Figure 2D–H), and GGPP treatment fully reversed the mRNA levels of SASP at 24 h as assessed by qPCR. Together, these results indicate statin-induced senescence in adipose tissue through GGPP depletion.

### 2.3. Ferroptosis Contributes to Atorvastatin-Induced Senescence in Adipose Tissue

**Figure 3:**
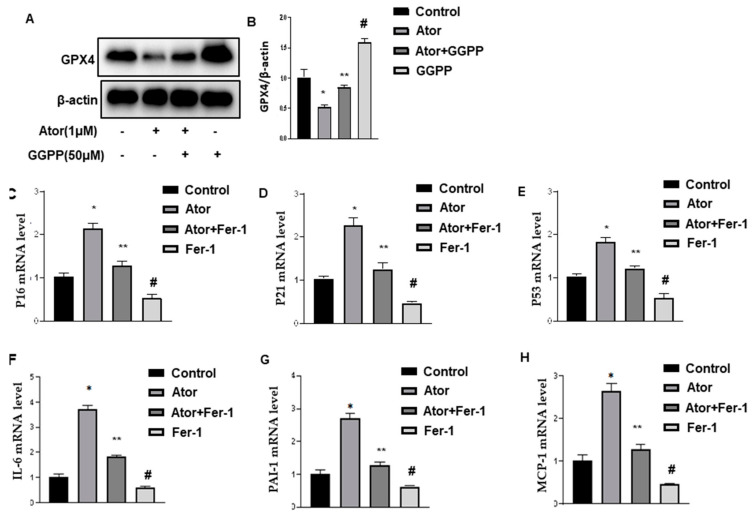

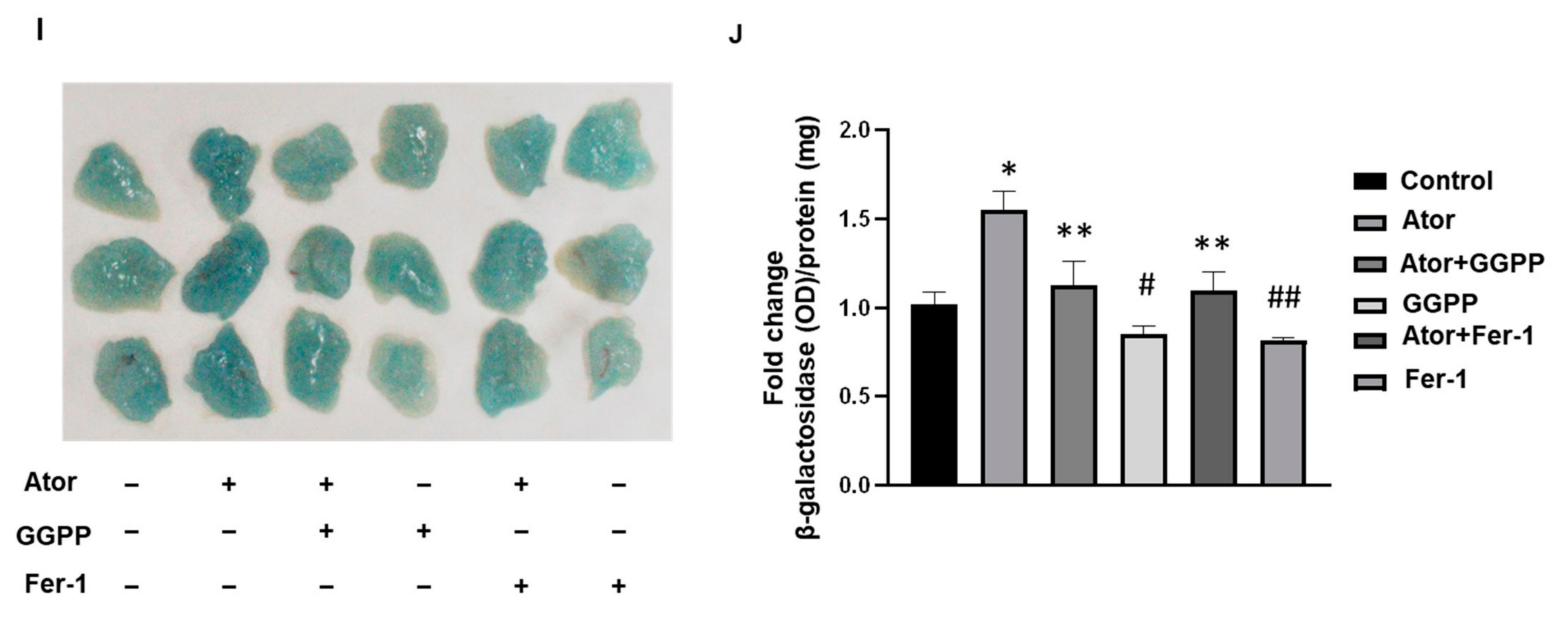
Ferroptosis contributes to atorvastatin-induced senescence in adipose tissue. (**A**,**B**). EAT explants were treated with atorvastatin (1 μM) plus supplementation with and without GGPP (50 μM). Representative immunoblot (**A**) and quantification (**B**) as indicated. All graphs correspond to the blot and represent densitometric analyses of 3 independent experiments. * *p * < 0.05 vs. Control; ** *p* < 0.05 vs. Ator; ^#^ *p* < 0.05 vs. control, Ator, and Ator + GGPP. (**C**–**E**). EAT explants were treated with atorvastatin (1 μM) plus ferrostatin-1 (Fer-1, 8 μM) for 18 h, as indicated. qPCR analysis of total RNA isolated from EAT for P16 (**C**), P21 (**D**), and P53 (**E**) mRNAs, respectively. Data were normalized to the amount of 18S mRNA and expressed relative to the corresponding control. *n* = 6 per group. * *p* < 0.05 vs. control. ** *p* < 0.05 vs. Ator; ^#^ *p* < 0.05 vs. Control, Ator, and Ator + Fer-1. (**F**–**H**). The mRNA levels of IL-6, PAI-1, and MCP-1 were quantified by qPCR. * *p* < 0.05 vs. control. ** *p* < 0.05 vs. Ator; ^#^ *p* < 0.05 vs. Control, Ator, and Ator + Fer-1. (**I**,**J**). Senescence was evaluated in terms of SA-β-galactosidase activity treated as indicated and expressed as the ratio of tissue protein (mg). *n* = 6 per group. * *p* < 0.05 vs. control. ** *p* < 0.05 vs. Ator; ^#^ *p* < 0.05 vs. Control, Ator, and Ator + GGPP; ^#^ *p* < 0.05 vs. Control, Ator, and Ator + Fer-1; ^#^^#^ *p* < 0.05 vs. Control. Data are mean ± SEM. Ator, atorvastatin; Fer-1, ferrostatin-1.

Adipose tissue explants were treated with atorvastatin for 18 h. The level of GPX4 was detected by Western blot. The results showed that atorvastatin significantly decreased the expression of GPX4 compared with control (Figure 3A,B). The supplementation of GGPP reversed atorvastatin-reduced GPX4 expression. Interestingly, GGPP alone exhibited a significant increase in the level of GPX4, which is likely related to obesity-reduced GPX4 in HFD mice [26].

To assess whether ferroptosis contributed to atorvastatin-induced senescence in adipose tissue, we treated these explants with ferrostatin-1 (Fer-1, 8 μM), a potent and selective inhibitor of ferroptosis. As shown in Figure 3C–E, Fer-1 treatment inhibited atorvastatin-induced cell cycle markers, the expression of P16, P21, and P53 (Figure 3C–E), and SASP-specific markers, including IL-6, PAI-1, and MCP-1 by qPCR in adipose tissue explants (Figure 3F–H). Fer-1 treatment alone led to a significant reduction of senescence, likely related to obesity-induced ferroptosis in HFD mice [26].

Next, SA-β-Gal staining was performed to monitor senescence. Atorvastatin increased SA-β-Gal activity (Figure 3I). The supplementation of GGPP partially blocked atorvastatin-induced SA-β-Gal activity in adipose tissues explants. Similarly, Fer-1 treatment exhibited the inhibitory effect. Interestingly, either GGPP or Fer-1 treatment alone displayed a similar inhibition profile. These observations suggested that ferroptosis contributes to atorvastatin-induced senescence in adipose tissue.

### 2.4. Atorvastatin Causes Lipid Peroxidation in Adipose Tissue

**Figure 4:**
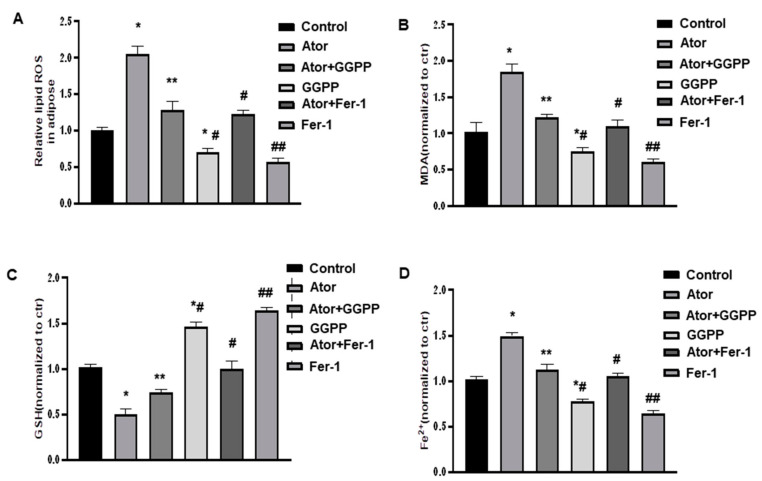
Atorvastatin causes lipid peroxidation in adipose tissue. (**A**). EAT explants were treated with atorvastatin (1 μM) plus either supplementation with and without GGPP (50 μM) or Fer-1 (8 μM) for 18 h, as indicated. The levels of ROS production in EAT were measured by ROS assay kit following instruction. (**B**–**D**). The levels of MDA, GSH, and Fe^2+^ in EATs were measured by following instructions. Data were expressed relative to the corresponding control. *n* = 6 per group. * *p* < 0.05 vs. control. ** *p* < 0.05 vs. Ator; *^#^ *p* < 0.05 vs. Control, Ator, and Ator + GGPP; ^#^ *p* < 0.05 vs. Ator; ^##^ *p* < 0.05 vs. Control, Ator, and Ator + Fer-1. Data are mean ± SEM. Ator, atorvastatin; Fer-1, ferrostatin-1.

We further investigated the effects of Fer-1 on oxidative stress and ferroptosis in adipose tissue. As shown in Figure 4A,B, atorvastatin exhibited significantly elevated ROS and MDA levels and reduced GSH levels compared with those in the control group. However, supplementation of GGPP markedly reversed these effects of atorvastatin on oxidative stress marker levels. Moreover, Fer-1 treatment displayed similar results (Figure 4C). Furthermore, as shown in Figure 4D, Fe^2+^ levels were significantly increased following atorvastatin, but either GGPP or Fer-1 treatment significantly reduced this atorvastatin-induced increase in Fe^2+^ levels in adipose tissue explants. Especially, either GGPP or Fer-1 treatment displayed strong effects when compared with control group. These results suggest that GGPP and Fer-1 treatment can alleviate atorvastatin-induced lipid peroxidation and suppress ferroptosis in adipose tissues.

### 2.5. Ferroptosis Is Not Involved in Atorvastatin-Induced GGPP Depletion in Adipose Tissue

**Figure 5:**
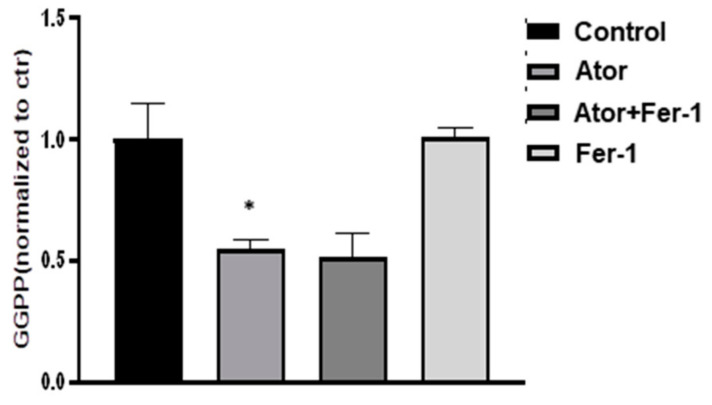
Ferroptosis is not involved in atorvastatin-induced GGPP depletion in adipose tissue. EAT explants were treated with atorvastatin (1 μM) plus Fer-1 (8 μM) for 18 h, as indicated. GGPP levels were measured by ELISA following the instruction. Data were expressed relative to the corresponding control. *n* = 6 per group. * *p* < 0.05 vs. control. Data are mean ± SEM. Ator, atorvastatin; Fer-1, ferrostatin-1.

The production of intermediates of the mevalonate pathway, such as GGPP, is critical for cell growth and apoptosis [27]. We therefore evaluated the effect of Fer-1 on atorvastatin-induced GGPP depletion in adipose tissue. As shown in Figure 5, atorvastatin treatment promoted GGPP depletion, and this reduction in GGPP content was not affected by Fer-1 treatment. In addition, Fer-1 treatment alone did not affect GGPP content in adipose tissue explants, suggesting that ferroptosis is not involved in atorvastatin-induced GGPP depletion in adipose tissues.

## 3. Discussion

In this study, we found that atorvastatin could induce senescence in adipose tissue, and supplementation with GGPP restored atorvastatin-induced senescence. Furthermore, the treatment of Fer-1, a selective ferroptosis inhibitor, restored atorvastatin-induced senescence. Our data suggest a novel mechanism for statin-induced senescence in adipose tissue.

GPX4 is positively related to inflammatory effects in adipocytes, and impaired GPX4 activity caused lipid peroxidation and expression of inflammatory cytokines such as TNF-α, interleukin 1β (IL-1β), IL-6 and the IL-8 homologue CXCL1 [28]. In this study, we found that treatment of atorvastatin significantly decreased GPX4 expression compared with control in adipose tissue, indicating that atorvastatin could evoke the pro-inflammatory effects in adipose tissue. Indeed, our results showed that atorvastatin treatment induced the expression of SASP-specific markers, including IL-6 and MCP-1, by qPCR.

GGPP is an endogenous regulator of adipocyte function and is an essential role in mediating adipocyte survival [29,30]. Henriksbo et al. reported that atorvastatin activates NLR family pyrin domain-containing 3 (NLRP3) and caspase-1/IL-1β inflammasome responses and impairs insulin-stimulated adipocyte lipogenesis [31]. In addition, atorvastatin mediated reductions in GGPP levels are partly responsible for impaired insulin signaling [31]. Thus, the mechanism underlying new-onset T2DM induction is associated with GGPP depletion by statin treatment. The isoprenoids used for prenylation are responsible for cholesterol generation and can be blocked by statins [32]. Recent study showed that isoprenoids required for protein prenylation were sufficient to prevent atorvastatin-mediated defects in insulin signaling in adipocytes [31]. We found that the treatment with atorvastatin significantly promoted GGPP depletion in adipose tissue. Supplementation with GGPP abrogated atorvastatin-induced senescence, suggesting that the senescent effects of atorvastatin are due to GGPP depletion. Importantly, GGPP alone showed significant inhibition in the levels of P16, P21, and P53 mRNA. We do not fully understand the exact mechanism. One possibility might be obesity-induced senescence in HFD mice [25].

Previous studies have shown that statins could downregulate the mevalonate pathway and block the biosynthesis of cellular isoprenoids, which are responsible for the synthesis of GPX4 [33,34]. There is no experimental evidence demonstrating the link between GGPP and GPX4. In our experiments, statin treatment significantly increased the proportion of SA-β-Gal staining and SASP gene markers expression, including PAI-1, MMP3, CD68, IL-6, MCP-1, and IGF-1, whereas ferroptosis inhibitor Fer-1 exhibited opposite effects. The crosstalk between ferroptosis and senescence remains poorly defined. Our results indicated that supplementation of GGPP could restore statin-downregulated GPX4 expression, and Fer-1 treatment exhibited the inhibitory effects on statin-induced senescence. Thus, ferroptosis may be a form of statin-induced senescence. Further studies are necessary to investigate the association between GPX4 and ferroptosis in adipose tissues.

Ferroptosis has been shown to be involved in various physiopathological processes linked to the iron-dependent accumulation of lipid ROS and oxidative stress. This study is the first to describe the relationship between statin and ferroptosis in adipose tissues. By treatment of atorvastatin, the levels of ROS, Fe^2+^, and MDA were increased, whereas the level of GSH was decreased. A recent study inducted that atorvastatin exposure causes excessive iron content, ROS production, and lipid peroxidation accumulation in muscular cells [35]. On the other hand, Simvastatin treatment reduced ROS production induced by cholesterol in the kidney cortical collecting duct cell line [36]. The discrepancy in metabolic characteristics may be related to individual statin types in different studies [35,37,38]. At the same time, Fer-1 could reverse the effects of atorvastatin on ROS production, Fe^2+^, MDA, and GSH, indicated that the excessive production of ROS might be important to occur ferroptosis in adipose tissue. Future studies were required to determine the relationships between statin, oxidative stress, and senescence in adipose tissue.

In the current study, atorvastatin is associated with lipid peroxidation product, MDA, via the GGPP in adipose tissue. We demonstrated that the ferroptosis inhibitor Fer-1 did not affect atorvastatin-induced GGPP depletion. However, supplementation of GGPP could reverse the effects of atorvastatin on lipid peroxidation, in which ferroptosis is regulated by lipid peroxidation. We believe that supplementation of GGPP protects the cells against ferroptosis by inhibiting lipid peroxidation.

**Figure 6:**
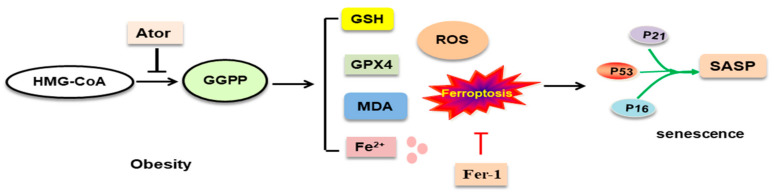
Schematic illustration of atorvastatin-induced GGPP depletion promoted ferroptosis-related senescence in adipose tissue. Fer-1, ferrostatin-1; GPX4, glutathione peroxidase 4; GSH, glutathione; ROS, reactive oxygen species.

In conclusion, the current study demonstrated, for the first time, that ferroptosis played an essential role in atorvastatin-induced senescence (P16, P21, and P53) and SASP-related pro-inflammatory cytokines (PAI-1, MMP3, CD68, IL-6, MCP-1, and IGF-1) in adipose tissue, and the supplementation of GGPP reversed atorvastatin-induced senescence, SASP, and lipid peroxidation. Atorvastatin induced levels of ROS, Fe^2+^, and GSH, and regulated GPX4 expression in adipose tissues (Figure 6). Our study was limited to dissecting the relationship between GGPP, ferroptosis, and senescence in adipose tissue. Further animal and cellular experiments are required to determine the roles of protein prenylation in statin-induced cell senescence in adipose tissue [31,32,39]. However, we have made the important and intriguing observations that either supplementation of GGPP or ferroptosis inhibition is required for reversing atorvastatin-induced senescence.

## 4. Materials and Methods

### 4.1. Reagents

Atorvastatin, cerivastatin, fluvastatin, and simvastatin, were purchased from Gödecke/Parke-Davis (Freiburg, Germany). Ferrostatin-1 (#S7243) was from Selleck Chemical (Houston, TX, USA). GGPP was obtained from GlpBio (Montclair, NJ, USA). The iron ion detection kit was purchased from Abcam. The malondialdehyde (MDA), and glutathione (GSH) kits are from Beyotime (Shanghai, China). ROS kit was purchased from bjbalb Inc. (Beijing, China).

### 4.2. Animals

C57BL/6J mice were obtained from Chengdu Gembio Inc. (Chengdu, China). The study was approved by the Ethics Committee of Southwest Medical University (Project identification code: 2020YJ0340). Eight-week-old male C57BL/6J mice were fed a high fat diet (HFD) (TP2330055A; fat calories 60%, carbohydrate calories 25%, and protein calories 15%; Research Diet, Trophic Animal Feed High-tech Co., Ltd., Nantong, China) for 16 weeks.

### 4.3. Experimental Design

Mice were sacrificed by cervical dislocation. As described previously [22,23], epidydimal adipose tissues (EAT) were isolated and minced into ~5-mg pieces in DMEM containing 10% FBS. After 2 h of incubation, 50 mg of small pieces were placed in serum-free DMEM and exposed to 1 µmol/L atorvastatin for 18 h, and 0.1% DMSO served as a control. In specific experiments, EAT explants were also treated with GGPP (50 μM; GlpBio), or ferrostatin-1 (Fer-1, 8 μM) [40], and added to the culture medium at the same time as was atorvastatin. Group animal size was *n* = 6–8 per group. The exact group size is specially described in the Figure legends.

### 4.4. Quantitative PCR (qPCR)

EAT was isolated and total RNA was extracted using TRIzol reagent (Invitrogen, Carlsbad, CA, USA). P16, P21, P53, PAI-1, MMP3, CD68, IL-6, MCP-1, and IGF-1 expression were measured using qPCR. RNA samples were pre-treated with deoxyribonuclease I (Invitrogen Life Technologies, Carlsbad, CA, USA), and a PrimeScriptTM RT Reagent Kit (Takara, Japan) was used to synthesize cDNA according to the manufacturer’s recommendations. qPCR was analyzed using SYBR^®^ RT-qPCR kit (Takara, Japan). Amplification was performed on an ABI PRISM 7700 cycler (Applied Biosystems, Foster City, CA, USA). Fold changes in gene expression were calculated using the 2^−ΔΔCT^ method. The values are shown as the mean ± SEM. All primers used in this study are listed in Supplemental Table S1.

### 4.5. Senescence-Associated-β-Galactosidase (SA-β-gal) Assay

The SA-β-gal activity was measured using Senescence Assay Kit (ST429, Beyotime) according to the manufacturer’s instruction. Briefly, EAT explants were incubated in ONPG at room temperature for 12 h and then stained with the staining mixture at 37 °C without CO_2_ overnight. Subsequently, EAT explants were observed and visualized under a light microscope (Zeiss, HAL 100, Berlin, Germany). The values were normalized to total protein levels assessed with a bicinchoninic acid (BCA) protein assay (Pierce).

### 4.6. Tissue ROS Levels

EAT was isolated, lysed, and the total amount of ROS was determined using the dihydroethidium (DHE) probe according to the manufacturer’s instructions. All values were normalized to total cellular protein, determined using a BCA assay, and expressed as intensity/mg protein. Data are expressed as the fold change relative to the control group.

### 4.7. Fe^2+^, MDA, and GSH Levels

For Fe^2+^ level measurement, EATs were isolated, homogenized in iron assay buffer and centrifuged at 16,000× *g* for 10 min. An iron chromogen Ferene S was added into samples. Each sample was further incubated with the iron probe at 37 °C for 60 min. For MDA, and GSH assay, EATs were isolated, homogenized, and lysed. Samples were centrifuged at 10,000× *g* for 10 min, and the supernatants were collected. MDA and GSH levels in the supernatant of EATs were evaluated. The MDA, Fe^2+^, and GSH kits were performed according to the manufacturer’s instructions. The values for the levels of MDA, Fe^2+^, and GSH in adipose tissue explants, were measured using a microplate reader at the absorption wavelengths of 532, 593, and 412 nm.

### 4.8. Immunoblotting

EATs were isolated, homogenized in lysis buffer and centrifuged at 16,000× *g* for 10 min. The supernatants were obtained. Equal amounts of EAT lysates were subjected to SDS-PAGE and transferred to polyvinylidene difluoride membranes by electroblotting. After blocking, the membranes were incubated with antibodies directed against GPX4 (Cell signaling). Secondary antibody was horseradish-peroxidase (HRP)-conjugated goat IgG raised against IgG (Cell signaling). Blots were developed with ECL substrate (Pierce). IMAGE J software was used to quantify band intensity.

### 4.9. ELISA

EATs were isolated, homogenized in lysis buffer and centrifuged at 16,000× *g* for 10 min. The supernatants were obtained, and GGPP concentrations were measured using commercially available ELISA kits (Shanghai Win-win Biotechnology Co., Ltd., Shanghai, China) according to the manufacturer’s protocol. GGPP values were calculated by plotting absorbance at 450 nm. Experimental values were compared with standard values. Data are expressed as the fold change relative to the control group.

### 4.10. Statistical Analysis

Data are presented as mean ± standard error of the mean. Experimental groups were compared by the two-tailed Student’s *t*-test or one-way analysis of variance (ANOVA).

## Data Availability

The data presented in this study are available on request from the corresponding author.

## Supplementary Materials

The following supporting information can be downloaded at: https://www.mdpi.com/article/10.3390/nu14204365/s1, Table S1: Primers used in qPCR.
