# Supplementary material for: Statin-Induced Geranylgeranyl Pyrophosphate Depletion Promotes Ferroptosis-Related Senescence in Adipose Tissue
Source: Nutrients. 2022 Oct 18;14(20):4365. doi: 10.3390/nu14204365 (PMC9607568; doi:10.3390/nu14204365)
Supplement: Supplementary file 1 [file nutrients-14-04365-s001.zip › nutrients-1939768-supplementary.pdf]

Table S1. Primers used in qPCR

Gene		Forward	Reverse
18S		CCTGGATACCGCAGCTAGGA	GCGGCGCAATACGAATGCCCC
P21		ACTTCCTCTGCCCTGCTGC	GGTCTGCCTCCGTTTTCG
P16		ATGGAGTCCGCTGCAGACAG	ATCGGGGTACGACCGAAAG
P53		CCCCAGGATGTTGAGGAGTT	TTGAGAAGGGACAAAAGATGACA
MCP-1		ATGCAGGTCCCTGTCATG	GTTCACTGTCACACTGGTCA
CD68		CTAGTCCAAGGTCCAAGGGG	TCCCTGGACCTTGGTTTTGT
MMP3		CCTCTATGGACCTCCCACAGAATC	GTGCCAATGCCTGGAAAGTTC
PAI-1		CTATGGTGAAACAGGTGGACTT	GAACTTAGGCAGGATGAGGAG
IL-6		CACATGTTCTCTGGGAAATCG	TTGTATCTCTGGAAGTTTCAGATTGTT
